# Evaluation of Marine Brown Algae and Sponges from Brazil as Anticoagulant and Antiplatelet Products

**DOI:** 10.3390/md9081346

**Published:** 2011-08-10

**Authors:** Laura de Andrade Moura, Fredy Ortiz-Ramirez, Diana Negrao Cavalcanti, Suzi Meneses Ribeiro, Guilherme Muricy, Valeria Laneuville Teixeira, Andre Lopes Fuly

**Affiliations:** 1 Department of Molecular and Cellular Biology, Institute of Biology, Federal Fluminense University, Niteroi, 24020-141, RJ, Brazil; E-Mail: lauravivimel@gmail.com; 2 Department of Marine Biology, Institute of Biology, Federal Fluminense University, Niteroi, 24020-141, RJ, Brazil; E-Mails: faortizr@hotmail.com (F.O.-R.); dn.cavalcanti@gmail.com (D.N.C.); valerialaneuville@gmail.com (V.L.T.); 3 Department of Invertebrates, Federal University of Rio de Janeiro, National Museum, 20940-040, RJ, Brazil; E-Mails: suzimr@yahoo.com.br (S.M.R.); muricy@mn.ufrj.br (G.M.)

**Keywords:** antithrombotic, bioprospecting, brown algae, marine sponges, natural products

## Abstract

The ischemic disorders, in which platelet aggregation and blood coagulation are involved, represent a major cause of disability and death worldwide. The antithrombotic therapy has unsatisfactory performance and may produce side effects. So, there is a need to seek molecules with antithrombotic properties. Marine organisms produce substances with different well defined ecological functions. Moreover, some of these molecules also exhibit pharmacological properties such as antiviral, anticancer, antiophidic and anticoagulant properties. The aim of this study was to evaluate, through *in vitro* tests, the effect of two extracts of brown algae and ten marine sponges from Brazil on platelet aggregation and blood coagulation. Our results revealed that most of the extracts were capable of inhibiting platelet aggregation and clotting measured by plasma recalcification tests, prothrombin time, activated partial thromboplastin time, and fibrinogenolytic activity. On the other hand, five of ten species of sponges induced platelet aggregation. Thus, the marine organisms studied here may have molecules with antithrombotic properties, presenting biotechnological potential to antithrombotic therapy. Further chemical investigation should be conducted on the active species to discover useful molecules for the development of new drugs to treat clotting disorders.

## 1. Introduction

Hemostasis is a physiological process that involves platelet aggregation and blood coagulation. The coagulation system is divided into initiation and augmentation phases, and both lead to generation of thrombin, that is the pivotal enzyme responsible for formation of a fibrin net that prevents hemorrhage [1]. Platelets are equally important to form thrombus. After their activation by agonists (collagen, adenosine diphosphate (ADP) or thrombin), they contribute by amplifying the blood coagulation system [2]. If uncontrolled, coagulation may produce vascular disturbs, such as venous thromboembolism, cardiovascular disturbs or pulmonary embolism. These pathologies in fact, contribute to increases in mortality and morbidity in the world and have become a public health problem [3]. Actually, they represent the main cause of death, even more than cancer [4]. Therefore, safe and more efficient anticoagulants should be designed or discovered. In literature and in practice, a variety of drugs for managing thrombotic disorders have been used, including vitamin K antagonists, direct thrombin inhibitors, oral anticoagulants (apixaban, dabigatran and rivaroxaban), and recently a pentasaccharide. Heparin and warfarin are among the drugs associated with serious medication errors and are also considered as high-alert medications [5,6]. Antiplatelet drugs are also given to yield the greatest benefits, mainly to those patients at high thrombotic risk. These drugs may interfere with platelet adhesion, aggregation or activation, thus controlling the role of platelets in amplifying the thrombotic process [7]. Although these therapies have proven benefits, they have important limitations and may produce side effects, such as bleeding, thrombocytopenia, gastric disorders, narrow therapeutic index and window, drug or dietary interactions as well as sometimes requiring monitoring [3,8]. In fact, antithrombotic therapies may induce mild or even more severe symptoms. Because of this, researchers are looking for molecules with satisfactory antiplatelet and/or anticoagulation effects, but with no or low side effects [9]. Bioactive natural molecules are good candidates for such purposes [10–12]. As known, marine organisms produce a large amount of products, often so called secondary metabolites. They display ecological functions such as defense against predators, competitors and herbivores, prevention of biofouling [13,14] and some of them have been reported as having important pharmacological proprieties [15,16], such as antiviral [17–19], antilonomic [20], antiophidic [21], cytotoxic, antituberculosis [22], anti-inflammatory, antiangiogenic, antiadhesive [23] and anticoagulant [24]. Moreover, a large number of sulfated polysaccharides with antithrombotic effects have been isolated from marine algae [23,25–28] as well as from other natural sources; such as from lichens [29,30] or from *Passiflora nitida* leaves [31]. The inhibitory mechanism of action of these is still under investigation, but some speculate that molecules bind directly to thrombin and factor Xa, antithrombin and/or heparin cofactor II [32,33]. So, the purpose of this work was to investigate the effect of extracts from marine organisms from the Brazilian coast upon coagulation and platelet aggregation.

## 2. Results and Discussion

### 2.1. Effects on Platelet Aggregation

**Figure 1.**
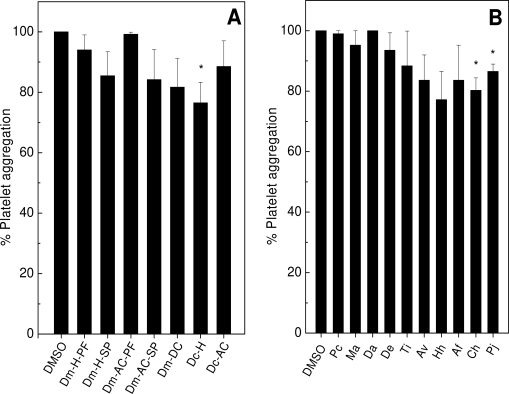
Effect of algae or sponges on platelet aggregation: Platelet rich plasma (PRP) was preincubated with dimethylsulfoxide (DMSO) (0.9% v/v, final concentration) extracts of algae (Panel **A**) or sponges (Panel **B**) for 2 min at 37 °C under stirring. Then, adenosine diphosphate (ADP) (15 μM) was added to induce platelet aggregation. One hundred percent of platelet aggregation was obtained with supramaximal concentrations of the agonist in presence of DMSO after 6 min. of reaction. Data are expressed as means ± SEM of three individual experiments (*n* = 2). Asterisk used when *p* < 0.05 compared to DMSO.

The effect of extracts from algae or sponges on platelet aggregation and coagulation was studied; and it was shown that both marine organisms inhibited platelet aggregation or prevented plasma coagulation. Firstly, the effect of algae or sponges upon platelet aggregation was evaluated and they did not inhibit collagen-induced platelet aggregation on platelet rich plasma (PRP) (data not shown). On the other hand, the extracts of alga Dc-H (Figure 1A) or the sponges Ch and Pc (Figure 1B), inhibited the aggregation induced by ADP around 20%. ADP and collagen bind to different receptors in platelet membrane and trigger platelet aggregation by different intracellular pathways [34,35]; and this fact may explain why algae or sponges inhibited only the ADP-induced aggregation, but not the collagen one. The extracts of algae or sponges did not induce aggregation on PRP (data not shown).

**Figure 2.**
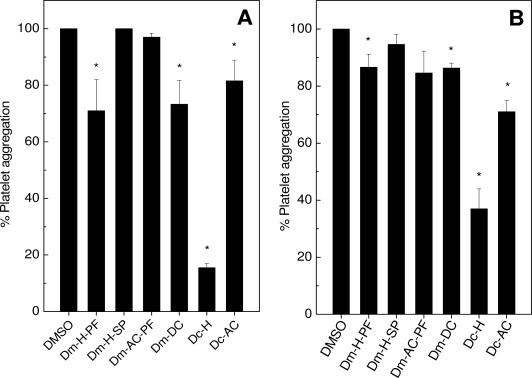

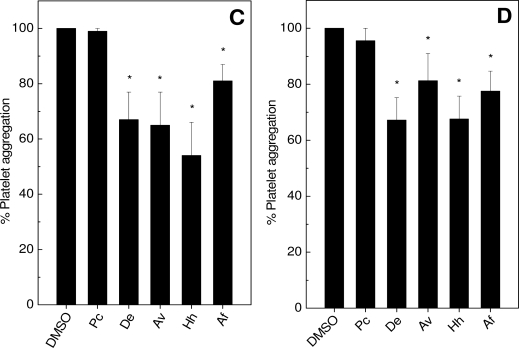
Effects of algae or sponges on washed platelet aggregation. The extracts of algae (50 μg/mL) or sponges (100 μg/mL) were preincubated with WP for 2 min at 37 °C under stirring, and then 0.4 U/mL thrombin (Panel **A**, **C**) or 16 μg/mL collagen (Panel **B**, **D**) was added to induce aggregation. One hundred percent of platelet aggregation was obtained with supramaximal concentrations of the agonists in presence of DMSO (0.9% v/v, final concentration) after 6 min of reaction. Data are expressed as means ± SEM of three individual experiments (*n* = 2). Asterisk used when *p* < 0.05 compared to DMSO.

Then, the effect of algae or sponges upon aggregation of washed platelets (WP) was investigated (Figure 2). The extracts of alga Dm-H-PF and Dm-DC inhibited 30% the aggregation induced by thrombin (Figure 2A) and 15% induced by collagen (Figure 2B); while Dc-H inhibited aggregation around 85% (Figure 2A) and 60% (Figure 2B). Dc-AC inhibited 20% and 35% aggregation induced by thrombin (Figure 2A) or collagen (Figure 2B), respectively. In fact, a more marked inhibitory effect on aggregation induced by thrombin or collagen could be observed for the extract of alga Dc-H (Figure 2A,B). Differences in the inhibitory profile may be explained by the fact that thrombin and collagen do not share the same receptors [36]. The experiments above were performed by incubating extracts of algae or sponges for 2 min with platelets. But when the algae Dc-H, Dm-H-PF, Dc-AC and Dm-DC were incubated with WP for 10 min, the inhibitory effect of them on platelet aggregation induced by collagen or thrombin was doubled (data not shown). The extracts of Dm-H-SP and Dm-Ac-PF did not inhibit aggregation of WP induced by thrombin (Figure 2A) or collagen (Figure 2B), even with 10 min incubation (data not shown). Four of ten sponges (De, Av, Hh and Af) inhibited aggregation of WP induced by thrombin (Figure 2C) or collagen (Figure 2D) after 2 min of incubation. Curiously, a 10% reduction on the inhibitory effect was observed when the sponges De, Hh or Af were incubated with platelets for 10 min (data not shown). The other two sponges (Pc, Av) did not inhibit aggregation either at 2 min or 10 min incubation. The extract of Pc did not interfere with platelet aggregation induced by both agonists (Figures 2C,D).

Regardless of the extract of sponge tested, a similar inhibitory pattern (from 50% to 80%) was observed for collagen or thrombin. As seen, most of algae and sponges did not inhibit platelet aggregation on PRP (Figure 1), in contrast to a pronounced inhibitory effect on WP (Figure 2). These results may suggest that blood plasma may somehow interfere in the inhibitory effect of extracts. Although the extracts inhibit platelet aggregation, they were not able to prevent shape-change which is at an early stage of platelet activation (data not shown). This dichotomy can be explained, since different receptors are involved in both processes. As known from literature, sugar rich-molecules can inhibit platelet aggregation [37–39]. However, none of the extracts from algae or sponges inhibited ristocetin-induced aggregation, except for alga Dc-H in which a 20% inhibition was observed (data not shown). So, the mechanism of action extracts seemed not to be the same as the sugars-enriched molecules. The inhibitory effect on aggregation of Dc-H, differed from other extracts of algae or sponges as it was concentration-dependent with an Inhibitory Concentration (IC_50_) of 56 μg/mL for collagen and 47 μg/mL for thrombin (data not shown). Surprisingly, the extracts of Ma, Da, Ti, Ch and Pj induced aggregation only when challenged with WP (data not shown), even though they have not induced aggregation on PRP. This result clearly shows the interference of plasma components in the extract-platelet interactions, since somehow molecules in plasma would bind to sponges’ metabolites, preventing them from inducing aggregation; this was the case of PRP. The proaggregating mechanism of action is still under investigation.

### 2.2. Effects on Coagulation

A large number of bioactive molecules with anticoagulant activity have been described in marine organisms. These molecules are regularly produced from their primary or secondary metabolism, including polysaccharides [28] or diterpenes [8,21]. So, the influence of the extracts of algae (Table 1) or sponges (Table 2) on coagulation was evaluated using four *in vitro* methods: (1) the recalcification time (RT); (2) prothrombin time (PT); (3) activated partial thromboplastin time (aPTT); and (4) fibrinogen clotting (FC). None of the extracts from algae or sponges exerted a pro-coagulant activity (data not shown). However, most of them inhibited coagulation, but with different potencies. The extracts of algae did not delay coagulation on the recalcification test at 5 or 10 min of incubation, but Dm-AC-PF and Dc-AC did delay PT and aPTT times, respectively (Table 1). The *in vitro* anticoagulant effect of such algae suggests that the former may have molecules that act in the extrinsic pathway (initiation) of the coagulation system, while the latter acts in the intrinsic one (augmentation). The higher anticoagulant effect of algae was observed upon fibrinogen clotting elicited by thrombin (Table 1). It is well known that thrombin generates fibrin through cleavage fibrinogen and induces platelet aggregation, and these two effects are mediated by two distinct sites, a catalytic site and a pharmacological one. So, we may speculate that algae extracts could also inhibit the enzymatic activity of thrombin. To test it, we evaluated the ability of algae to inhibit hydrolysis of S-2238 that is a specific substrate for thrombin. However, none of the extracts prevented hydrolysis by thrombin (data not shown); suggesting that the anticlotting effect of algae upon fibrinogen might not be associated with thrombin activity or that algae do not bind to the catalytic site of thrombin.

Moreover, the extracts of sponges also inhibited clotting (Table 2). It seems that sponges were more effective than algae, since they inhibited coagulation in the four methods (Table 2). The extracts of Ti, Ch and Pj doubled coagulation time in each of the four methods; but, Ti was the most effective. Only the extracts of sponges Da, De and Hh did not delay coagulation in the recalcification test (Table 2). As in the algae tests, the extracts of sponges did not inhibit hydrolysis of S-2238 (data not shown).

Concerning the extracts of algae, the collection site and the type of extract may have influenced the results, mainly because of the geographical variation of each species and the chemical composition of extracts. It is worthwhile to mention that the polarity of solvents may also influence the potency of some pharmacological effects, since different molecules can be extracted [40]. The extracts evaluated in this work contain nonpolar molecules, such as diterpenes, fatty acids or sterols. As can be seen, the extracts of *D. menstrualis* prepared in ethyl acetate from Búzios (Dm-AC-PF) and *D. ciliolata* in hexane from Angra dos Reis (Dc-H) presented the most expressive anticoagulant activity. The extract of Ti had the best anticoagulant effect among sponges.

## 3. Experimental Section

### 3.1. Material

Collagen (Type I), ristocetin and adenosine diphosphate (ADP) were purchased from Chrono Log Corporation, dimethylsulfoxide (DMSO) and human thrombin from Merck. Chromogenic substrate, H-D-Phe-pipecolyl-Arg-pNA.2HCl (S-2238) came from Chromogenix. All the others reagents were of the best grade available.

### 3.2. Marine Organisms

The air-dried algae (Table 3) and the frozen-dried sponge (Table 4) were collected in regions of Brazilian coast by free or scuba diving and extracted three times with appropriate solvent at room temperature (25 °C) for 24 h (Tables 3 and 4). After filtration, the solvent was evaporated under reduced pressure, yielding a crude residue. An aliquot of each extract was weighted, aliquoted and frozen. The extracts were dissolved in DMSO in order to perform the biological assays, and right after assays, extracts were stored at 4 °C. A voucher specimen of each sponge has been deposited at National Museum of the Federal University of Rio de Janeiro (MN-UFRJ), Rio de Janeiro, Brazil. Each species of algae was deposited at the Herbarium of State University of Rio de Janeiro (UERJ), RJ, Brazil and authenticated by Dr. Joel Campos de Paula (UNIRIO). The experimental procedures employed here removed sugar, lipids and proteins, and extracted less polar components and nonpolar ones, as diterpenes, fatty acids and sterols.

### 3.3. Platelet Aggregation Assays

The procedure described by [41] was employed with some modifications. Platelet aggregation was measured turbidimetrically in an Aggregometer (Chrono Log model 490 2D, Havertown, USA) using Platelet-Rich-Plasma (PRP) or Washed-Platelets (WP). PRP (collected in citrate 0.38% w/v) and WP (collected in 5 mM EDTA) were obtained from whole blood of healthy volunteer donators and centrifuged at 380× *g* for 12 min at room temperature. The extracts of either algae or sponges were incubated with 250 μL PRP or WP for 2 min at 37 °C under stirring, and then platelet aggregation was triggered by adding platelet agonists (collagen, ADP, ristocentin or thrombin). Assays were performed at 37 °C in siliconized glass cuvettes with a total volume of 300 μL. Control experiments were performed using platelet agonists in the presence or in the absence of DMSO (0.9% v/v, final concentration). One hundred percent (100%) of aggregation was obtained with a supra maximal concentration of agonists 6 min after the addition of agonist, whilst 0% of aggregation was the light transmittance of PRP or WP alone (base line). The Inhibitory Concentration (IC_50_) was determined as the amount of extract (μg/mL) able to inhibit 50% of platelet aggregation induced by agonists.

### 3.4. Clotting Assays

Four *in vitro* clotting assays were performed using a Multichannel Coagulometer (Amelung, model KC4A), as listed below.

Recalcification Time (RT): The extracts of algae or sponges were incubated with 200 μL of a pool of normal human plasma (collected in citrate 3.8% v/v) for 10 min at 37 °C. Then, CaCl_2_ (12.5 mM final concentration) was added to the mixture and clotting time recorded.

Prothrombin Time (PT) and Activated Partial Thromboplastin Time (aPTT) tests: Both assays were performed according to the manufacturer instructions (Soluplastin and aPTTest elagic acid, Wiener lab). For PT assays, extracts were incubated with 50 μL of human plasma during 5 min at 37 °C and then 100 μL of pre-warmed thromboplastin with calcium were added to the mixture to start clotting. For aPTT assay, extracts were mixed with 100 μL of human plasma and 100 μL of aPTT reagent cephalin plus kaolin for 5 min at 37 °C. The reaction was started by the addition of CaCl_2_ (12.5 mM, final concentration).

Fibrinogen clotting (FC): The extracts of algae and sponges were incubated for 10 min at 37 °C with 200 μL of commercial fibrinogen (2 mg/mL, final concentration). After this, clotting was triggered by adding thrombin (1.0 U/mL).

### 3.5. Hydrolytic Activity upon Chromogenic Assay

The hydrolysis of the chromogenic substrate S-2238 (used for thrombin-like enzymes) was monitored by using a Thermomax Microplate reader (Molecular Devices, Menlo Park, CA, USA). The extracts of algae or sponges were incubated for 10 min at 37 °C, and then reaction was triggered by adding S-2238 (0.1 mM, final concentration). The reaction was monitored at A_405nm_ during 20 min at 37 °C. Positive control experiments were performing by adding thrombin (1.0 U/mL), instead of marine organisms.

### 3.6. Statistical Analyses

The results were expressed as means ± standard error (SEM) obtained with an indicated number of experiments performed. The statistical significance of differences among experimental groups and control groups were evaluated using Student *t* test with *p* value <0.05.

## 4. Conclusions

In this work, we screened extracts from Brazilian algae and sponges having the capacity to interfere with platelet aggregation and coagulation and, thereby, observed that some extracts inhibited coagulation and platelet aggregation. Further chemical work should be done to identify which specific bioactive principle in algae or sponges is responsible for the anticoagulant effect and also to understand the structure-function relationship involved in such phenomena. Moreover, this work also shows the importance of bioprospecting studies with the rich marine biodiversity along the Brazilian coast, looking for natural compounds with pharmacological properties or biotechnological potential which could be used in the development of new clinical drugs. Up to now, it is very difficult to address advantages of algae or sponges over existing anticoagulant therapies or their role in thrombotic processes. However, we can infer some favorable points that are worth further studying and looking for molecules from marine organisms with an anticoagulant effect: (1) actually, there is more interest in developing therapeutic agents from a non-mammalian source in order to diminish side effects (such as hemorrhage), risk of contamination with foreign bodies or pathogenic substances; (2) marine products could also be useful to help to design new structure-based anticoagulant drugs.

## Figures and Tables

**Table 1.** Effect of algae extracts on clotting times. For RT, the extracts of algae (50 μg/mL) were preincubated with plasma and then CaCl_2_ was added to trigger coagulation. For PT, extracts (100 μg/mL) were incubated with plasma and then thromboplastin was added to induce coagulation. For aPTT, extracts (100 μL/mL) were incubated with plasma plus cephalin then CaCl2 was added to trigger coagulation. For FC, extracts (100 μg/mL) were incubated with fibrinogen and then thrombin (1 U/mL) was added to induce coagulation. Results are expressed as means ± SEM of two individual experiments (*n* = 4). Asterisk used when *p* < 0.05 compared to NaCl or DMSO (1% v/v). See Table 3 for species names.

**Samples**	**Clotting times (s)**			
	**RT**	**PT**	**aPTT**	**FC**
NaCl	122.8 ± 16	21.7 ± 0.3	32.1 ± 1.2	26.4 ± 0.5
DMSO	149.7 ± 3.3	25.6 ± 0.2	38.8 ± 0.8	34.1 ± 1.4
Dm-H-PF	158.3 ± 11	24.5 ± 0.2	38.7 ± 2.0	46.8 ± 1.3 *
Dm-H-SP	156.2 ± 8.3	25.1 ± 0.4	39.3 ± 1.3	47.3 ± 1.6 *
Dm-AC-PF	172.3 ± 13	29.9 ± 0.6 *	40.5 ± 1.1	86.4 ± 2.6 *
Dm-AC-SP	155.6 ± 3.2	24.7 ± 1.0	39.1 ± 0.7	65.3 ± 3.5 *
Dm-DC	171.6 ± 14	23.3 ± 0.2	40.2 ± 0.5	66.7 ± 0.8 *
Dc-H	177.4 ± 13	27.3 ± 0.2	38.7 ± 2.4	42.7 ± 0.9 *
Dc-AC	166.1 ± 4.1	28.9 ± 0.2	46.1 ± 2.5 *	78.4 ± 11 *

**Table 2.** Effect of the extracted sponges on clotting time. For RT, the extracts (50 μg/mL) were preincubated with plasma and then CaCl_2_ was added to trigger coagulation. For PT, extracts (100 μg/mL) were incubated with plasma and then thromboplastin was added to induce coagulation. For aPTT, the extracts (100 μL/mL) were incubated with plasma plus cephalin then CaCl_2_ was added to trigger coagulation. For FC, the extracts (100 μg/mL) were incubated with fibrinogen and then thrombin (1.0 U/mL) was added to induce coagulation. Results are expressed as means ± SEM of two individual experiments (*n* = 4). Asterisk used when *p* < 0.05 compared to NaCl or DMSO (1% v/v). See Table 4 for species names.

**Samples**	**Clotting times (s)**			
	**RT**	**PT**	**aPTT**	**FC**
NaCl	124.3 ± 7.2	21.7 ± 0.3	32.1 ± 1.2	25.2 ± 0.1
DMSO	160.6 ± 11.5	22.7 ± 0.2	34.8 ± 1.3	34.4 ± 0.4
Pc	203.5 ± 4.7 *	27.4 ± 1.0 *	48.5 ± 1.4 *	86.6 ± 5.8 *
Ma	227.0 ± 8.0 *	27.1 ± 0.7 *	54.8 ± 2.0 *	99.8 ± 9.6 *
Da	210.6 ± 17.1	27.9 ± 1.2 *	52.3 ± 1.7 *	88.9 ± 6.9 *
De	197.2 ± 10.6	26.3 ± 0.4 *	54.1 ± 1.2 *	101.6 ± 6.0 *
Ti	246.0 ± 9.3 *	27.3 ± 0.8 *	64.2 ± 1.7 *	176.2 ± 4.7 *
Av	239.3 ± 14.8 *	27.1 ± 0.6 *	58.0 ± 1.6 *	94.4 ± 5.4 *
Hh	185.6 ± 10.1	23.9 ± 0.6	40.6 ± 1.1 *	76.6 ± 3.1 *
Af	234.6 ± 13.6 *	25.7 ± 0.5 *	45.2 ± 2.2 *	75.9 ± 5.4 *
Ch	296.3 ± 29.0 *	27.5 ± 0.4 *	53.2 ± 2.8 *	92.6 ± 8.1 *
Pj	278.6 ± 6.5 *	26.3 ± 0.5 *	52.2 ± 1.0 *	107.1 ± 5.0 *

**Table 3.** Extracts of algae studied with their solvent, collection site, voucher specimen and code.

**Algae**	**Solvent**	**Collection Site**	**Voucher**	**Code**
*Dictyota menstrualis*	Ethyl Acetate	Arquipélago de São Pedro e São Paulo, RN (00°55′N–29°21′W)	11006	*Dm*-AC-SP
*Dictyota menstrualis*	Ethyl Acetate	Praia do Forno Armação de Búzios, RJ (22°45′S–41°52′W)	84815	*Dm*-AC-PF
*Dictyota menstrualis*	Hexane	Arquipélago de São Pedro e São Paulo, RN (00°55′N–29°21′W)	11006	*Dm*-H-SP
*Dictyota menstrualis*	Hexane	Praia do Forno Armação de Búzios, RJ (22°45′S–41°52′W)	84815	*Dm*-H-PF
*Dictyota menstrualis*	Dichloromethane	Praia Rasa Armação de Búzios, RJ (22°45′S–41°52′W)	10017	*Dm*-DC
*Dictyota ciliolata*	Hexane	Praia Vermelha Angra dos Reis, RJ (23°00′S–44°19′W)	10415	*Dc*-H
*Dictyota ciliolata*	Ethyl Acetate	Praia Vermelha Angra dos Reis, RJ (23°00′S–44°19′W)	10415	*Dc*-AC

**Table 4.** Extracts of sponges studied with their solvent, collection site, voucher specimen and code.

**Sponge**	**Solvent**	**Collection Site**	**Voucher**	**Code**
*Petromica citrina*	Dichloromethane	Arquipélago das Cagarras Rio de Janeiro, RJ (23°01′S–43°13′W)	14537	Pc
*Mycale angulosa*	Acetone	Praia da Baleia Angra dos Reis, RJ (23°01′S–44°14′W)	14529	Ma
*Chondrosia* sp.	Acetone	Praia da Baleia Angra dos Reis, RJ (23°01′S–44°14′W)	14551	Ch
*Dysidea etherea*	Acetone	Praia da Baleia Angra dos Reis, RJ (23°01′S–44°14′W)	14525	De
*Desmapsamma anchorata*	Acetone	Ilha do Bonfim Angra dos Reis, RJ (23°01′S–44°19′W)	14520	Da
*Amphimedon viridis*	Acetone	Ilha do Bonfim. Angra dos Reis, RJ (23°01′S–44°19′W)	14517	Av
*Tedania ignis*	Acetone	Praia de Tarituba Paraty, RJ (23°02′S–44°35′W)	14522	Ti
*Hymeniacidon heliophila*	Acetone	Praia de Itaipu Niterói, RJ (22°58′S–43°02′W)	14528	Hh
*Aplysina fulva*	Acetone	Praia do Forno Arraial do Cabo, RJ (22°57′S–42°00′W)	13554	Af
*Polymastia janeirensis*	Acetone	Praia do Forno Arraial do Cabo, RJ (22°57′S–42°00′W)	14512	Pj
